# Magnetic NiFe thin films composing MoS_2_ nanostructures for spintronic application

**DOI:** 10.1038/s41598-022-14060-w

**Published:** 2022-06-13

**Authors:** Mahdi Yousef Vand, Loghman Jamilpanah, Mohammad Zare, Seyed Majid Mohseni

**Affiliations:** 1 Materials Engineering, Faculty of Engineering, University of Tehran, Tehran, Iran; 2 Department of Physics, Shahid Beheshti University, Evin, Tehran, 19839 Iran

**Keywords:** Engineering, Physics, Materials science

## Abstract

We demonstrate a nanostructure layer made of Ni_80_Fe_20_ (permalloy:Py) thin film conjugated MoS_2_ nano-flakes. Layers are made based on a single-step co-deposition of Py and MoS_2_ from a single solution where ionic Ni and Fe and MoS_2_ flakes co-exist. Synthesized thin films with MoS_2_ flakes show increasing coercivity and enhancement in magneto-optical Kerr effect. Ferromagnetic resonance linewidth as well as the damping parameter increaseed significantly compared to that of the Py layer due to the presence of MoS_2_. Raman spectroscopy and elemental mapping is used to show the quality of MoS_2_ within the Py thin film. Our synthesis method promises new opportunities for electrochemical production of functional spintronic-based devices.

## Introduction

Recent promising achievements in spintronics specially in magnetic thin films conjugated to two-dimensional (2D) materials has made this topic interesting for fundamental studies to explore their important role in the future spintronic-based memory and computing devices^1–5^. The core deriving fundamental phenomenon in such structures is the spin–orbit interaction (SOI)^6,7^. To benefit from SOI in spintronic devices, materials with high spin–orbit coupling (SOC), mostly heavy metals like Pt and Ta^8^ are used in devices in contact to magnetic thin films. Also, due to the recent developments in the field of 2D materials, special focus is put into implementing 2D materials with their intriguing properties instead of those heavy metals^9,10^ with high SOC. Many studies have demonstrated the successful usage of transition metal dichalcogenides (TMDCs) in contact to ferromagnetic thin films to enhance the SOI, induce surface anisotropy, etc.^11,12^. We have recently demonstrated that the magnetic anisotropy can be tuned by MoS_2_ on the surface of Py thin films^13^ and also predicted interfacial anisotropy can be changed in Co/black-phosphorene^14^. Here, we alternatively demonstrate the magnetic properties of Ni_80_Fe_20_ change by embodiment of MoS_2_ thin flakes. This shows the whole single ferromagnetic thin film to possess SOC-induced intrinsic magnetic properties.

Fabrication of thin films for spintronics devices based on physical techniques such as sputtering and thermal evaporation have shown the best performance^15,16^. Besides, electrodeposition method has established to be very promising in producing spin valves with very high number of layer repetitions (above 100 repeated layers^17^) and also functional nanowires for spin caloritronic devices^18–20^. Although it should be mentioned that electrodeposition lacks the ability to provide ultra-thin films without voids or making multilayers of diverse types of materials in a single growth^21^. The implementation of 2D materials in contact to ferromagnetic thin films has been challenging^22,23^ and such structures are made by transferring the as-made 2D layers on the ferromagnetic layers^24^. In addition to their multi-step fabrication method, the materials contacts are poor that hitherto limits their reproducibility and scalability^25^. Therefore, developing new fabrication method for making heterostructure of 2D materials/ferromagnetic layers is demanded to achieve higher yield and functionality.

In this work, we use electrodeposition method for fabrication of Py magnetic films and present the co-electrodeposition of MoS_2_ thin flakes with ionic elements of the solution. The Raman spectroscopy indicates successful embodiment of thin MoS_2_ flakes inside the grown magnetic film. The magnetic properties of the layer with MoS_2_ flakes show prominent differences with bare ferromagnetic layer including higher magnetic coercivity and damping parameter which are directly related to the enhancement in SOC of the medium. Our results indicate that our fabrication method has resulted in a good proximity between the MoS_2_ and the magnetic material for inducing SOC in the ferromagnet. Our method has the possibility of being used for growth of gradients or multilayers of the investigated material through control of the growth conditions like applied growth voltage/current.

## Experimental section

### Exfoliation of MoS_2_

**Figure 1:**
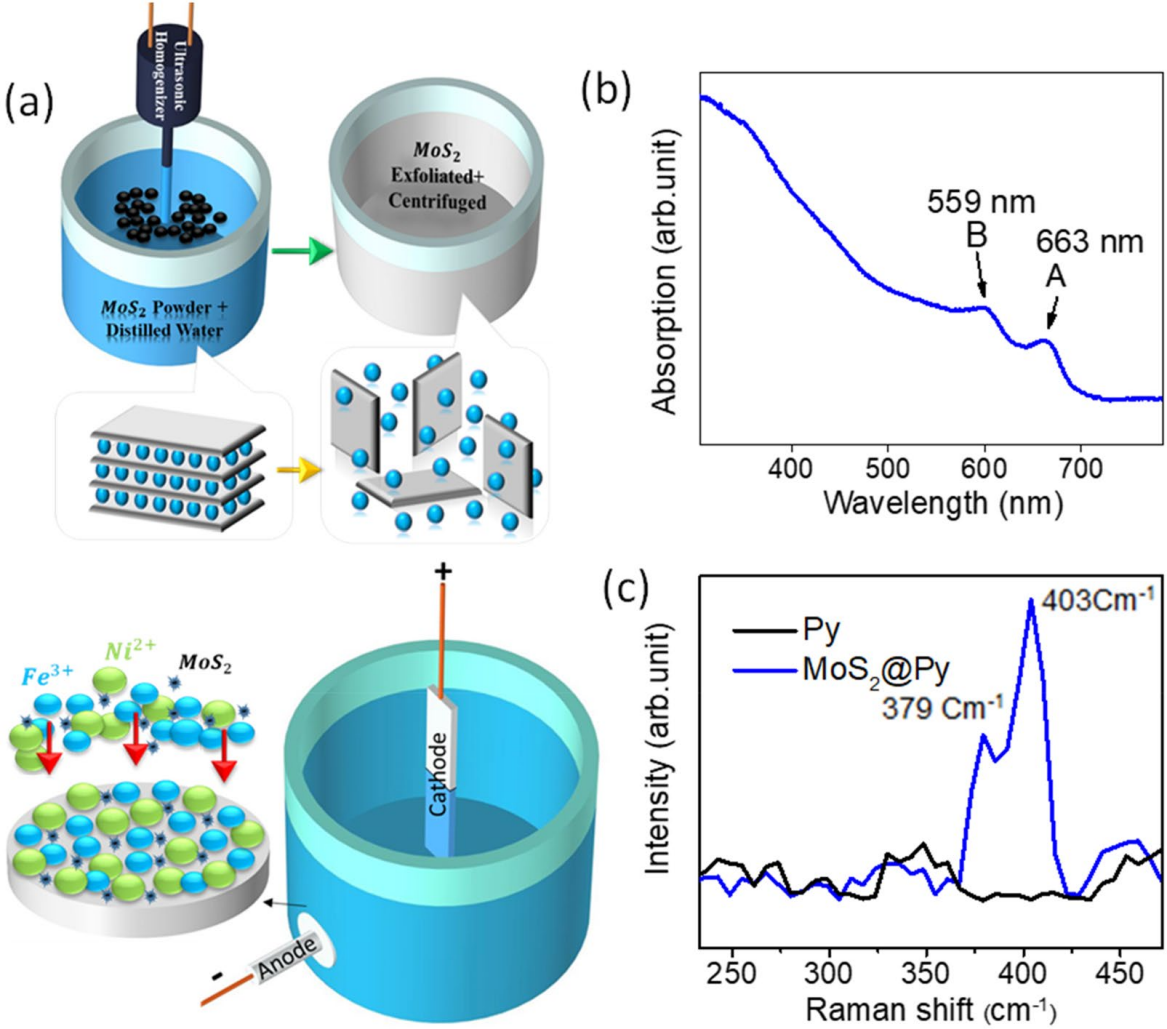
(**a**) Schematic of the MoS_2_ exfoliation by probe sonication (above) and the schematic of electrodeposition of samples (below). (**b**) The UV–Visible absorption of the exfoliated MoS_2_ and (**c**) the Raman spectrum of Py and MoS_2_@Py samples.

Exfoliation of MoS_2_ was done for a 1 g MoS_2_ powder (Aldrich, 99%, < 2 μm) in 100 ml distilled water, equivalent to 10 g l^−1^ concentrations of MoS_2_ (Fig. 1a). The MoS_2_ powder was exfoliated for 4 h using a sonication probe unit equipped with a long step horn tip. In order to avoid excessive heating, the probe of sonic was set to operate 0.7 s and rest for 0.3 s and also an ice-water bath was used during the exfoliation. The resulting solution was centrifuged for 30 min at 200 rpm to remove non-exfoliated particles.

### Electrodeposition

Electrodeposition was done from two different solutions. First solution for electrodeposition of the Py sample had the composition of 0.4 M NiSO_4_.6H_2_O, 0.04 M FeSO_4_.7H_2_O, and 0.4 M H_3_BO_3_ (originally prepared from Merk) in distilled water. 200 ml of this solution was used for the electrodeposition. For the electrodeposition of the Py conjugated MoS_2_ sample (MoS_2_@Py), second solution was prepared as the following. First a 100 ml of the same solution with doubled molarities was prepared and then a 100 ml of the exfoliated MoS_2_ solution was added to it. This way the final molarities of Ni and Fe ions is similar in both the solutions. Si substrates (single side polished surface) were cut by 1.5 × 1.5 cm slices. In order to clean surface of the Si from native oxide, they were dipped in 10% HF (hydrochloric acid) solution for 45 s, then washed by ethanol, acetone and distilled water respectively, and dried by air pump. Then the Si substrates were immediately transferred to the electrodeposition cell to prevent further surface oxidation. A two-electrode cell configuration with a DC current source was used for the electrodepositions. A 2 × 1 cm platinized Si was used as the anode and Si substrate as the cathode. The Py and MoS_2_@Py samples were electrodeposited by applying a direct voltage of 10 V at room temperature during 120 and 150 s, respectively.

### Characterization

UV–Visible (Perkin Elmer, Lambda25) and Raman spectroscopy (Teksan) were carried out at room temperature. Surface was probed via atomic force microscopy (AFM, nanosurf) measurement. Energy dispersive X-ray spectroscopy (EDX) and elemental mapping were measured by through field emission scanning electron microscope (FESEM, Hitachi). Magnetic hysteresis loops were measured by longitudinal magneto-optical Kerr effect (MOKE), with a 632 nm laser light (a home-made setup). Ferromagnetic resonance (FMR) measurements were performed by a home-made field modulation lock-in technique at the frequency range of 2–20 GHz (for details see supporting information).

## Results

Schematic of the exfoliation condition is depicted in Fig. 1a where the force of water molecules results in exfoliation of the MoS_2_ powder into thin flakes. To characterize the quality of the exfoliated MoS_2_ in water, UV–Visible absorption measurement was used. Result of this measurements can be seen in Fig. 1b. The A and B peaks at 559 and 663 nm respectively are the characteristic of few layer MoS_2_ dispersions. After solution preparation and electrodeposition of the layers (Fig. 1a below) Raman characterization is used to see if the MoS_2_ flakes are imbedded in the body of the Py layer. Figure 1c presents the Raman spectrum for Py and MoS_2_@Py samples. Raman peaks at 379 and 403 cm^−1^ clearly show the presence of MoS_2_ flakes in the electrodeposited layer^26^. The bare Py sample shows no peak in its Raman spectrum because it has a metallic nature.

**Figure 2:**
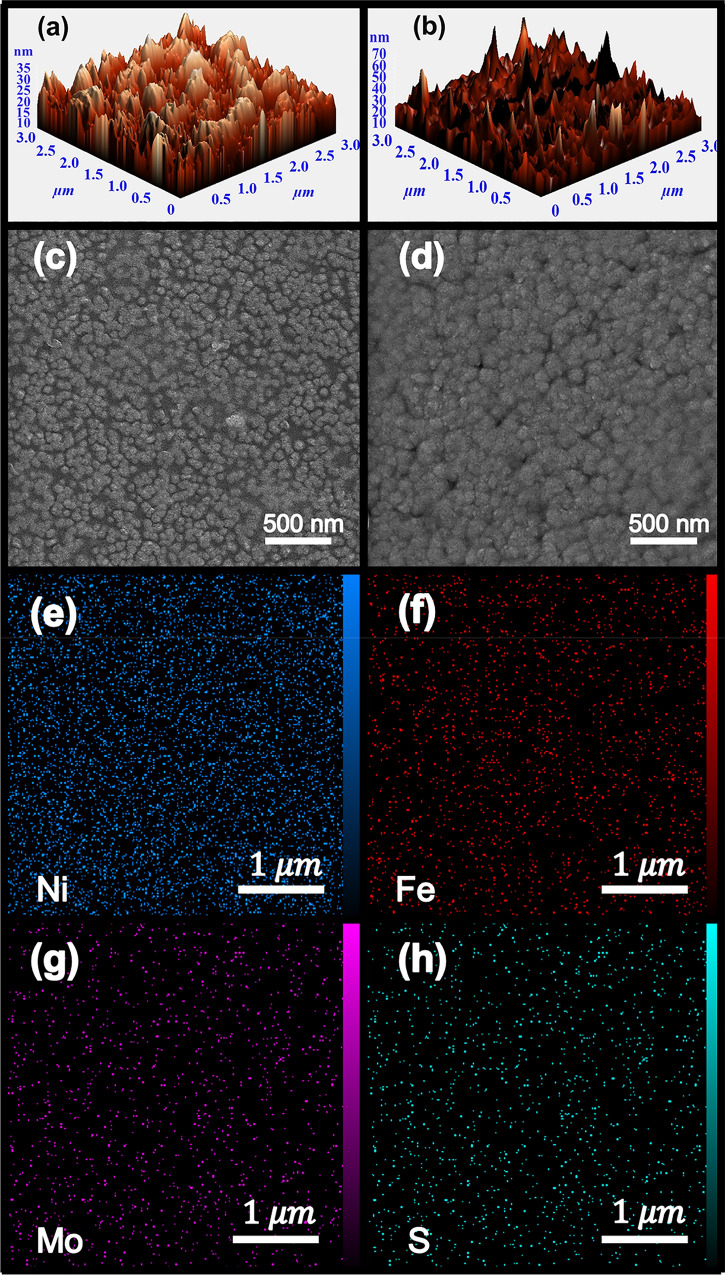
AFM measurement of (**a**) Py and (**b**) MoS_2_@Py samples. The FESEM images of (**c**) Py and (**d**) MoS_2_@Py samples. (**e**) and (**f**) are the EDX mapping of Py sample and (**g**) and (**h**) the EDX mapping of the MoS_2_@Py sample.

The surface topography of the Py and MoS_2_@Py samples has been observed with AFM and presented in Fig. 2a and b, respectively. The AFM images show that both samples have a similar surface structure with an increased mean surface roughness for the MoS_2_@Py sample to 45 nm from the 20 nm mean surface roughness of the Py sample. Also, FESEM characterization of the Py and MoS_2_@Py samples has been performed and results are presented in Fig. 2c and d, respectively. It can be seen that both the samples have a granular structure with an increased grain size for the MoS_2_@Py sample. Also cross sectional FESEM images of the samples are presented in supporting information which show thicknesses of ~ 50 and ~ 100 nm for Py and MoS_2_@Py samples (~ 10% error), respectively. The observed higher thickness of the MoS_2_@Py samples is related to the partial space occupation by MoS_2_ and also the slightly higher electrodeposition time of this sample. Distribution of MoS_2_ in Py layer has been evidenced by EDX measurement. Figure 2e–h represents the EDX mapping of Ni, Fe, Mo and S elements where the uniform color distribution shows the uniform embodiment of MoS_2_. Also, the atomic ratio of Ni:Fe is 4:1.

**Figure 3:**
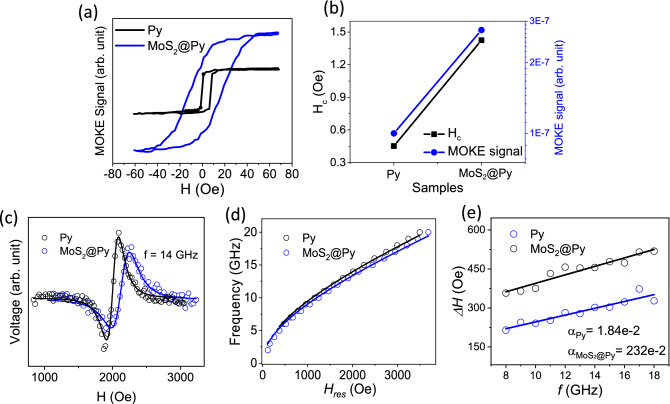
(**a**) MOKE signal measured for the samples and (**b**) comparison of the H_c_ and the MOKE signal intensity of the samples. (**c**) the FMR signal observed at f = 14 GHz, (**b**) the fitted *f* and H_res_ curve based on Kittel formula and (**c**) the data of FWHM versus *f* and their fitting based on the damping relation.

In continue, magnetic properties of the samples are investigated through the MOKE and FMR measurements. Longitudinal MOKE measurement results are presented in Fig. 3a showing that both the samples have an in-plane magnetic anisotropy. Two prominent differences are appeared in the MOKE signal of the samples. One is the much higher coercivity (H_c_) of the MoS_2_@Py sample which is depicted in Fig. 3b. The H_c_ for the Py sample is ~ 10 Oe and has increased to ~ 30 Oe for the MoS_2_@Py sample which is equivalent to a 300% increase. In the case of films, the magnetic anisotropy of ferromagnetic layers has been demonstrated to change by proximity to TMDC layered materials due to the d-d hybridization at the interface^11,13,14^. In the case of MoS_2_@Py sample, all interfacial directions between Py and MoS_2_ is possible which overall has resulted in the observed in-plane coercivity change. Generally, magnetic anisotropy is highly dependent on the SOC^27–29^ and by addition of MoS_2_ as a material with high SOC to the layer, changes in the magnetic anisotropy is expected. One should note that increase of the in-plane coercivity can result from the increase of thickness because of the emerging out of plane magnetic anisotropy component at higher thicknesses^30–34^. To see if the observed increase of H_c_ in our samples is due to the relatively higher thickness of the MoS_2_@Py sample we performed MOKE measurements for different thicknesses of Py layer and only a slight change in the H_c_ was observed (for the details see supporting information). Therefore, we conclude that the observed changes in the magnetic properties of Py is due to its proximity to MoS_2_. Also, the H_c_ of the samples can be affected by the grain size^35^ and the AFM and SEM images indicate a comparably bigger grain size for the MoS_2_@Py sample. But the totally different MOKE signal quality of the MoS_2_@Py sample, including the slope of the plot, indicates that presence of MoS_2_ is playing a crucial role in this increased H_c_.

The other observation from the MOKE signal is the much higher signal intensity of the MoS_2_@Py sample relative to Py sample which is increased by 275%. Two mechanism can play role in the observed MOKE signal increase for the MoS_2_@Py sample: (1) increase of the saturation magnetization (*M*_*s*_) and (2) increase of light interaction with matter^36,37^. Here the increase of the MOKE signal cannot be due to the increase of *M*_*S*_, as the *M*_*S*_ decreases for the MoS_2_@Py sample (See FMR section). Therefore, increase of the MOKE signal can be related to the increased interaction between light and the MoS_2_@Py sample. For the case of multilayered ferromagnet/TMDC heterostructures it has been reported that proper thickness, refraction index and incident angle, can form a cavity^38,39^. Here we achieved enhancement of the MOKE signal via composing MoS_2_ flakes with the magnetic layer. Enhancement of light-mater interaction also has been achieved via electrophoretic deposition of MoS_2_ nanostructures^40^. Simulation of the MOKE signal for the composed MoS_2_@Py sample are encouraged to obtain the optimized composing structure. Also, we do not ignore the possibility of the larger grain size in the MoS_2_@Py being responsible partially for the observed MOKE signal intensity increase. For determining the exact contribution of each parameter further experiments should be designed.

We have also measured the FMR characteristics of the samples to see how MoS_2_ can affect the magnetization dynamics of the Py layer. The spin dynamic response of the samples investigated at different constant microwave frequencies ranging from 2 to 20 GHz and by sweeping *H* from 0 to 4500 Oe. The observed FMR spectra were fitted with the derivative of Lorentzian function to determine the resonance field (*H*_*r*_) and FWHM (*∆H)* at each frequency. Figure 3c presents the measured FMR signal of the samples (dots) and their fit (solid line) at *f* = 14 GHz. The frequency dependence of *H*_*r*_ for the Py and MoS_2_@Py samples can be seen in Fig. 3d (dots). The solid lines in this figure are the fitted data by Kittel’s equation. Dependence of the FMR frequency on the external magnetic field for thin films assuming with infinite dimensions which are saturated in the plane can be described by Kittel formula^41^,

$$f_{r} = \frac{{{\upmu }_{0} \gamma }}{2\pi }\sqrt {\left( {H + H_{k} + M_{s} } \right)\left( {H + H_{k} } \right)}$$

where ${\upmu }_{0}$ is the permeability of the free space, $\gamma$ is the gyromagnetic ratio (28 GHz/Tesla), H is the external magnetic field and $H_{k}$ is the uniaxial anisotropy field which is negligible for Py films with low thickness. The resulted *M*_*S*_ from this fit are 10,448 and 9391 Oe for the Py and MoS_2_@Py samples, respectively. A decrease of about 10% in the *M*_*s*_ of the MoS_2_@Py sample is observed which can be understood via the nonmagnetic character of the MoS_2_ that results in a lower magnetization per unit volume. Also, both samples do not show magnetic anisotropy. By using the damping relation^42^, as the following:

$$\Delta H = \Delta H_{0} + \frac{4\pi \alpha f}{\gamma }$$

and fitting the data we can calculate the damping parameter of the samples. Here Δ*H*_*0*_ is the inhomogeneous broadening, and $\alpha$ is the Gilbert damping parameter. The fitting of the data based on this equation can be seen in Fig. 3e. The value of Δ*H*_*0*_ for Py and MoS2@Py samples are 114 and 231 Oe respectively. All the obtained parameters from the FMR data are presented in Table 1.

**Table 1:** The *α*, *M*_*s*_*, *$\gamma$, *H*_*k*_, *ΔH*_*0*_ obtained from the fitting of FMR data.

	Py	MoS_2_@Py
*α*	0.018	0.023
*M*_*s*_ (Oe)	10,448	9391
$\gamma$	0.0028	0.0028
*H*_*k*_ (Oe)	0	0
*ΔH*_*0*_ (Oe)	114	231

In the obtained results, we see a doubling of the damping coefficient in the MoS_2_@Py sample compared to the Py sample, which has increased from 0.018 to 0.023. There are many reports that show the coating of a nonmagnetic layer on a ferromagnetic layer can lead to the enhancement of the damping parameter^43–45^. Several mechanisms have been proposed for the enhancement of damping parameter in such bilayers. SOC and interfacial d-d hybridization cause the enhancement of the intrinsic damping, while extrinsic enhancement of the damping can arise from two-magnon scattering processes, due to roughness and defects at the interface region^46–48^. In the case of our sample, embodiment of MoS_2_ in the Py layer results in the increased interfaces between Py and MoS_2_ and therefore both intrinsic and extrinsic contribution can contribute in the observed increase of the damping parameter. Moreover, the coupling between a FM layer and an adjacent NM layer can enhance the effective damping of the magnetization precession via spin-pumping effect^44,49^. For example, many groups have recently demonstrated the generation of spin–orbit torque in devices made with the Py/WTe_2_^50^, CoFeB/MoS_2_ or WSe_2_^51^, and Py/MoS_2_^52^ and the reciprocal process (voltage generation from spin-pumping) in MoS_2_/Al/Co heterostructures^53^. In the case of our MoS_2_@Py sample we observe the phenomena via enhanced damping parameter in a relatively thick magnetic layer thanks to the embodiment of the MoS_2_ flakes inside the layer which gives a high contact surface area between MoS_2_ and Py.

## Conclusions

MoS_2_ was successfully embodied within the structure of the Py magnetic thin film by electrodeposition method. The layer with MoS_2_ flakes shows a higher magnetic coercivity and Gilbert damping parameter, indicating the proper bonding between the MoS_2_ and the magnetic material. In addition, the cavity of light in the MoS_2_@Py sample resulted in a three-fold increase of the MOKE signal which opens a pathway for the research on the optimization of MOKE sensors and also fundamental studies in the field. Due to the capability of applying our method for a large set of ferromagnetic/TMDC materials, there is a great potential for further development of functional spintronic and magnonic devices.

## Supplementary Information

Supplementary Information.
