# Supplementary material for: Magnetic NiFe thin films composing MoS2 nanostructures for spintronic application
Source: Sci Rep. 2022 Jun 13;12:9809. doi: 10.1038/s41598-022-14060-w (PMC9192644; doi:10.1038/s41598-022-14060-w)
Supplement: Supplementary file 1 — Supplementary Information. [file 41598_2022_14060_MOESM1_ESM.docx]

**Supplementary information for:**

**Magnetic NiFe thin films composing MoS_2_ nanostructures for spintronic application**

M. Yousef Vand, L. Jamilpanah, M. Zare, S.M. Mohseni^*^

Department of Physics, Shahid Beheshti University, Evin, 19839 Tehran, Iran

This file includes:

1- Details of the Ferromagnetic resonance (FMR) measurement

2- Thickness of the Py and MoS_2_@Py samples

3- Thickness dependent magneto-optical Kerr effect (MOKE) signal of Py

4- “Data availability” statements

**1- Details of the FMR measurement**

Figure S1a represents schematic of the FMR setup. Using a Sub-Miniature version A (SMA) connector, a radio frequency (RF) field at a fixed frequency and fixed output power of 16 dBm was applied to a 50 Ω micro-stripe line with 200µm width. The external DC (Direct Current) magnetic field (H) swept at different constant values of microwave frequency ranging from 5-20 GHz for the Py sample and 2-20 GHz for the MoS_2_@Py sample with step of 1 GHz. FMR measurements were done with the geometry of parallel H and normal RF signal to the plane of samples. Samples mounted face down in contact to the stripe line without any electrical contacts for RF excitation (Figure S1b). RF signals coming out from the samples was passed through a Schottky diode via another SMA connector for detection. Field modulation technique was implemented to influence the sensitivity with lock-in technique. An external low frequency (342 Hz) AC (Alternating Current) field was applied via a pair of Helmholtz coil to improve the signal to noise ratio. All equipment were connected to a PC through a GPIB to fully control the experiment. Figure S1c shows the schematic of the sample.

Figure S1: a) Schematic diagram of the FMR spectrometry using field modulation technique, b) mounted sample on the micro-strip line and c) the schematic of the sample structure.

**2- Thickness of Py and MoS_2_@Py samples:**

Figure S2 presents the cross section FESEM images of the Py (left) and MoS_2_@Py (right) samples. The thickness of the Py is about 50 nm and the thickness of the MoS_2_@Py is about 100 nm (~10% error).

**
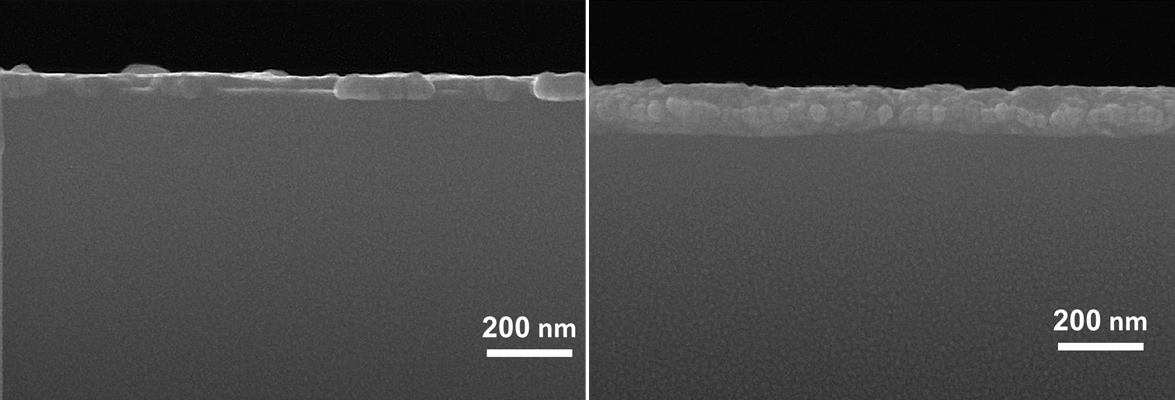
**

Figure S2: cross sectional FESEM images of Py (left) and MoS_2_@Py (right) samples.

**3- Thickness dependent MOKE signal of Py:**

Two bare Py samples with different thicknesses of ~50 (S1) and ~70 nm (S2) are coated to see if the much higher coercivity and MOKE signal of the MoS_2_@Py sample is due to the higher thickness of this sample. Cross-sectional FESEM image of sample S1 is shown in Figure S3a with the inset showing thickness of the two samples. Faraday’s law has been used to achieve different thicknesses of Py^1,2^. This law determines the thickness of layer that coated with electrochemical method^3^. The thickness (T) as a function of electrodeposition time (t) and current (i) can be described by:

$$T=\frac{\alpha\times i\times M\times t}{10^{-7}\times F\times S\times n\times\rho}$$

Where M is the molar mass of substance, F is the Faraday’s constant, S is the area of coated layer, n is the number of electrons involved in an electrode reaction, $\rho$ is the density of substance, and $\alpha$ is the return coefficient.

MOKE results of the two samples is presented in Figure S3b. as can be seen in this figure there is a very slight change observed in the MOKE signal of the two samples. This clearly indicates that the observed very high MOKE signal and coercivity for the MoS_2_@Py is due to the presence of MoS_2_.


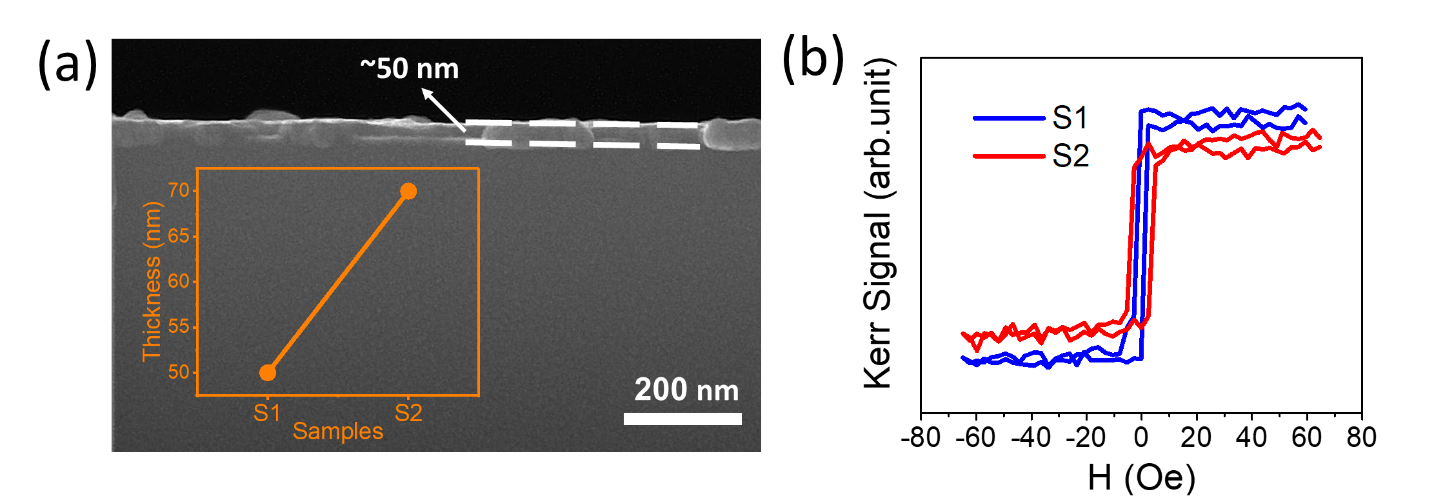


**Figure S3:** a) Cross sectional FESEM image of S1 sample. The inset shows the thickness of the two samples. b) The MOKE signal of the two electrodeposited S1 and S2 samples.

**4- “Data availability” statements:**

The datasets used and/or analyzed during the current study are available from the corresponding author on reasonable request.

**References**

1. Barker, D. & Walsh, F. C. Applications of Faraday’s Laws of Electrolysis in metal finishing. *Trans. Inst. Met. Finish.* **69**, 158–162 (1991).

2. Walsh, F. C. Overall rates of electrode reactions. Faraday’s Laws of Electrolysis. *Trans. Inst. Met. Finish.* **69**, 155–157 (1991).

3. Valizadeh, S., George, J. M., Leisner, P. & Hultman, L. Electrochemical synthesis of Ag/Co multilayered nanowires in porous polycarbonate membranes. *Thin Solid Films* **402**, 262–271 (2002).
